# Supplementary material for: Oral Health and Hygiene Practices from Baramulla District, Jammu and Kashmir, India: A Questionnaire-Based Cross-Sectional Observational Survey
Source: Healthcare (Basel). 2025 Feb 20;13(5):458. doi: 10.3390/healthcare13050458 (PMC11899622; doi:10.3390/healthcare13050458)
Supplement: Supplementary file 1 [file healthcare-13-00458-s001.zip › healthcare-3427610-supplementary.pdf]

# SUPPLEMENTARY

**Supplementary Table S1.** The various locations of the 10 Medical Blocks of Baramulla district from where our respondents belonged.

| | Medical Blocks | | | | | | | | | |
|---|---|---|---|---|---|---|---|---|---|---|
| | **Block 1** | **Block 2** | **Block 3** | **Block 4** | **Block 5** | **Block 6** | **Block 7** | **Block 8** | **Block 9** | **Block 10** |
| Locations | **Uri** | **Tangmarg** | **Sopore** | **Pattan** | **Rohama** | **Boniyar** | **Kunzer** | **Sheeri** | **Dangiwacha** | **Kreeri** |
| 1 | Isham | Drung | Dangaer-pora | Hamray | Nadihal | Now-shera | Manglo ra | Khwaja Bagh | Chatoosa | Nowpora |
| 2 | Nambla | Katipora | Harewan | Singhpora | Binner | Bijhama | | Kanth Bagh | Watergam | Doudh Bough |
| 3 | Gingal | Warpora | San-grama | Haigaam | Had-ipora | Limber | | Kalni Bagh | | Mulgam |
| 4 | | Namblinaar | Ladoora | | Chakloo | | | Ushkara | | Bulgam |
| 5 | | Ferozpora | Achabal | | Wanpora | | | Delina | | Wagoora |
| 6 | | | Rajpora | | Shitloo | | | Malpora | | |
| 7 | | | Arampora | | | | | Kanaspora | | |
| 8 | | | Zaingeer | | | | | Jahama | | |
| 9 | | | Seer Jagir | | | | | Jetti | | |
| 10 | | | | | | | | Jalsheeri | | |
| 11 | | | | | | | | Pachaar | | |
| 12 | | | | | | | | Old Town Baramulla | | |
| 13 | | | | | | | | Azadgunj | | |
| 14 | | | | | | | | Gutiyaar | | |
| 15 | | | | | | | | Kandiyaar | | |
| 16 | | | | | | | | Noor bagh | | |
| 17 | | | | | | | | Khanpora | | |
| 18 | | | | | | | | Fatehgarh | | |
| 19 | | | | | | | | Laridari | | |
| 20 | | | | | | | | Kitchama | | |
| 21 | | | | | | | | Katyanwali | | |
| 22 | | | | | | | | Drangbal | | |
| 23 | | | | | | | | Gunlista | | |
| 24 | | | | | | | | Fatehpora | | |
| 25 | | | | | | | | Kralhaar | | |
| 26 | | | | | | | | Chandoosa | | |
| 27 | | | | | | | | Heevan | | |
| 28 | | | | | | | | Janbazpora | | |
| 29 | | | | | | | | Singh Bagh | | |
| 30 | | | | | | | | Pothkaal | | |
| 31 | | | | | | | | Bungla Bagh | | |
| 32 | | | | | | | | Kaliban | | |
| 33 | | | | | | | | Rangwaar | | |
| 34 | | | | | | | | Posh Bagh | | |

**Supplementary Table S2**. Distribution of participants in various socio-economic groups based on the ration-cards type they possessed across various age groups.

| Age (years) | BPL | APL | PHH/ AAY/Others |
|---|---|---|---|
| 1–10 | 27 (43.55 %) | 24 (38.71 %) | 11 (17.74 %) |
| 11–20 | 37 (45.12 %) | 30 (36.59 %) | 15 (18.29 %) |
| 21–30 | 50 (34.25 %) | 69 (47.26 %) | 27 (18.49 %) |
| 31–40 | 43 (40.57 %) | 47 (44.34 %) | 16 (15.09 %) |
| 41–50 | 31 (51.67 %) | 24 (40.00 %) | 5 (8.33 %) |
| 51–60 | 8 (42.11 %) | 9 (47.37 %) | 2 (10.53 %) |
| 61 and above | 6 (46.15 %) | 5 (38.46 %) | 2 (15.38 %) |

* BPL: Below Poverty Line, APL: Above Poverty Line, PHH: Priority Households and AAY:Antyodya Anna Yojana.

**Supplementary Table S3a**. Distribution table of education level attained by participants across various age groups.

| Age (years) | Primary School | Middle School | High School | UG, Diploma | PG/Ph.D./Higher | Non-literate/Informal/Maqtab/Others |
|---|---|---|---|---|---|---|
| 1–10 | 47 (75.81 %) | 5 (8.06 %) | | | | |
| 11–20 # | 4 (4.88 %) | 27 (32.93 %) | 33 (40.24 %) | 15 (18.29 %) | 2 (2.42 %) | |
| 21–30 ** | 1 (0.68 %) | 10 (6.85 %) | 41 (28.08 %) | 61 (41.78 %) | 23 (15.75 %) | 8 (5.47 %) |
| 31–40 *** | 1 (0.94 %) | 17 (16.04 %) | 35 (33.02 %) | 14 (13.21 %) | 19 (17.92 %) | 18 (16.98 %) |
| 41–50 | 3 (5.00 %) | 13 (21.67 %) | 22 (20.75 %) | 7 (11.67 %) | 5 (8.33 %) | 10 (9.43 %) |
| 51–60 | 0 | 8 (42.11 %) | 4 (21.05 %) | 2 (10.53 %) | 1 (5.26 %) | 4 (21.05 %) |
| 61 and above | 1 (7.69 %) | 2 (15.38 %) | 2 (15.38 %) | 3 (23.08 %) | 2 (15.38 %) | 3 (23.08 %) |

* 10 children had not started school; ** 2 chose not to respond in this age group; *** 1 chose not to respond in this age group; # 1 chose not to respond in this age group.

**Supplementary Table S3b**. Distribution table of education level attained by male and female participants across various age groups.

| Age (years) | Primary School | | Middle School | | High School | | UG, Diploma | | PG/Ph.D./Higher | | Non-literate/Informal/Maqtab/Others | |
|---|---|---|---|---|---|---|---|---|---|---|---|---|
| | Male | Female | Male | Female | Male | Female | Male | Female | Male | Female | Male | Female |
| 1–10 | 18 | 29 | 3 | 2 | | | | | | | | |
| 11–20 # | 2 | 2 | 10 | 17 | 12 | 21 | 7 | 8 | 1 | 1 | | |
| 21–30 ** | 0 | 1 | 4 | 6 | 15 | 26 | 29 | 32 | 13 | 10 | 0 | 8 |
| 31–40 *** | 1 | 0 | 9 | 8 | 16 | 19 | 10 | 4 | 12 | 7 | 3 | 15 |
| 41–50 | 1 | 2 | 9 | 4 | 13 | 9 | 3 | 4 | 5 | 0 | 4 | 6 |
| 51–60 | 0 | 0 | 7 | 1 | 4 | 0 | 1 | 1 | 1 | 0 | 3 | 1 |
| 61 and above | 0 | 1 | 1 | 1 | 1 | 1 | 3 | 0 | 1 | 1 | 1 | 2 |

* 10 children had not started school; ** 2 chose not to respond in this age group; *** 1 chose not to respond in this age group; # 1 chose not to respond in this age group.
